# Dynamics of antibody engagement of red blood cells *in vivo* and *in vitro*

**DOI:** 10.3389/fimmu.2024.1475470

**Published:** 2024-11-28

**Authors:** Ryan P. Jajosky, Diyoly Ayona, Amanda Mener, Sean R. Stowell, Connie M. Arthur

**Affiliations:** Joint Program in Transfusion Medicine, Department of Pathology, Brigham and Women’s Hospital, Harvard Medical School, Boston, MA, United States

**Keywords:** alloimmunization, red blood cell, antibody, antigen, AMIS

## Abstract

Exposure to allogenic red blood cells (RBCs), either through pregnancy or transfusion, can result in alloimmunization, which can lead to severe hemolytic transfusion reactions and pregnancy complications. Passively administered antibodies can be used to prevent alloimmunization, where steric hindrance of allogeneic epitopes has been postulated as one mechanism whereby antibody engagement may prevent RBC alloimmunization. However, the dynamics of antibody engagement on the RBC surface has remained difficult to study. To examine this, we leveraged the HOD (HEL, OVA and Duffy) model system and Fcγ receptor knockout recipients to define the dynamics of antibody engagement of the Duffy antigen in the absence of RBC clearance or antigen modulation. Using this approach, the on-rate of antibody engagement of HOD RBCs was very similar *in vivo* and *in vitro*, with high levels of antibody binding observed within minutes of HOD RBC exposure. In contrast, the off-rate of HOD RBC bound antibody was relatively slow, with appreciable dissociation not being observed for an hour. However, the dynamics of antibody interactions with HOD changed significantly when antibody decorated HOD RBCs were exposed to free antibody. Despite the presence of prebound antibody, free antibody rapidly associated with HOD RBCs, with the rate of free antibody association observed being faster *in vivo* than *in vitro*. Importantly, antibody association and dissociation occurred in the absence of any appreciable changes in RBC clearance, antigen modulation or complement deposition, suggesting that differences in antibody levels observed reflected actual differences in the dynamics of antibody binding. These results suggest that while antibodies appear to be relatively static on the cell surface once bound, antibody engagement can be quite dynamic, especially in the face of free antibody in solution. These results not only have implications in the mechanisms of antibody-mediated immunosuppression, but also the potential use of other antibody-based approaches designed to prevent hemolytic transfusion reactions or target antigens *in vivo* in general.

## Introduction

Despite being a life-saving medical intervention, red blood cell (RBC) transfusion is not without risk. RBCs display numerous clinically significant alloantigens, including Duffy, KEL, and Kidd, that can become the target of alloantibody responses (1–3). Transfusion of RBCs positive for an alloantigen into an alloimmunized recipient can trigger a severe and potentially fatal hemolytic transfusion reaction (4–7). To mitigate this risk, pre-transfusion testing is conducted to identify potential incompatibilities between the donor unit and the recipient. When patients develop alloantibodies against multiple alloantigens or against a highly prevalent antigen, it can become challenging to procure compatible blood for future transfusions, directly increasing the likelihood of transfusion-related complications (2, 8, 9). As evidence of the challenges these alloantibodies can create, RBC alloimmunization is directly associated with increased morbidity and mortality rates, especially in transfusion-dependent patients (10, 11). The consequences of RBC alloimmunization are not limited to patients who require blood transfusion. The development of alloantibodies can also complicate pregnancies due to the potential for alloantibodies to bind to fetal RBCs, leading to hemolytic disease of the fetus and newborn (HDFN) (12, 13).

The development of alloantibodies against RBCs can occur through different mechanisms. Some, like anti-ABO antibodies, are naturally occurring and may be stimulated by exposure to environmental sources, ectopic expression of similar antigens within the host or completely independent of known antigen stimulation (14–17). In contrast, alloantibodies directed against most non-ABO alloantigens result from exposure to alloantigen-positive RBCs as a result of transfusion or pregnancy (2). While a variety of pathways may regulate the likelihood of alloimmunization following allogenic RBC exposure (17–27), the development of alloantibodies can form a barrier to future transfusion. Indeed, recipient recognition of allogenic RBCs can initiate an adaptive immune response that ultimately results in the development of alloantibodies capable of causing hemolytic transfusion reactions and HDFN (21, 28–30). Similar to adaptive immune responses in general, alloantibodies that form in response to RBC alloantigen exposure can persist or evanesce (31). Alloantibody evanescence, in particular, can pose risks to transfused recipients as testing strategies can miss evanescent alloantibodies, resulting in inadvertent alloantigen re-exposure following subsequent transfusion (32, 33). Alloantigen re-exposure places patients at risk for a recrudescent alloantibody response that can lead to delayed hemolytic transfusion reactions (DHTRs) (18, 33–37). These transfusion reactions can be accompanied by hyperhemolysis (38–41), which can be particularly life-threatening.

Following the initial recognition of RBC-induced alloimmunization and the consequences that maternal alloimmunization to the RhD antigen can have on the developing fetus, significant efforts have focused on understanding risk factors for pregnancy-associated alloimmunization. Early studies suggested that ABO incompatibility between the mother and fetus reduced the likelihood that an RhD-negative mother would develop alloantibodies following pregnancy with an RhD-positive fetus. These epidemiological findings, coupled with early work indicating that passive antibody administration could prevent *de novo* antibody formation following antigen exposure, implied that anti-A and anti-B antibodies may suppress the maternal immune response to fetal RhD-positive RBCs (42). Subsequent studies demonstrated that passive administration of IgG anti-RhD antibodies can prevent *de novo* antibody formation in the vast majority of RhD-negative women pregnant with an RhD-positive fetus. This strategy of administering anti-D immunoglobulin continues to be employed today to prevent anti-RhD-mediated HDFN.

Despite the success of AMIS in reducing alloimmunization rates to RhD during pregnancy or following transfusion, this approach is limited to the RhD antigen. HDFN arising from alloimmunization to other RBC alloantigens continues to occur without any current prevention options. One of the challenges associated with extending this therapeutic approach to additional alloantigens is an incomplete understanding regarding the overall mechanism whereby AMIS prevents alloantibody formation (43). Many competing hypotheses exist, some of which evoke RBC removal, antigen modulation in the absence of RBC clearance, engagement of inhibitory receptors, and steric hindrance of the target antigen (42–45). Recent studies using a variety of model systems have suggested that antibody engagement of RBCs can have divergent outcomes, with some antibodies enhancing alloimmunization, while others induce AMIS (46, 47). Variations in the ability of antibodies to induce antigen modulation, RBC clearance, both, or other mechanisms entirely may be responsible for these distinct outcomes (45, 46, 48–58).

While recent studies have begun to provide insight into possible mechanisms of AMIS, directly observing antibody engagement or inhibition of antigen recognition *in vivo* has remained challenging. This difficulty arises partly because antibodies can induce rapid RBC clearance or antigen modulation (45, 47, 54, 56, 57, 59, 60), making it hard to isolate the overall outcome of antibody interactions with target antigens alone *in vivo*. The concept of antibody-induced steric hindrance often suggests that following antibody binding, the dissociation rate is slow, precluding antigen recognition by masking the alloantigen epitope. This same approach has been proposed as a potential strategy to mask antigens and avoid hemolytic transfusion reactions in cases of RBC incompatibility, where incubation of RBCs with a non-functional antibody could conceal an antigen, rendering these RBCs insensitive to removal because of epitopes being blocked from antibody engagement. Consistent with this possibility, antibody engagement can mask epitope sites *in vivo (* 61), precluding the detection of distinct antigen determinants.

Whether antibody binding is relatively static *in vivo* due to a slow dissociation rate, thereby providing a possible explanation for AMIS or a strategy to prevent hemolytic transfusion reactions, remains incompletely understood. To investigate the dynamics of antibody equilibrium on the RBC surface in detail, we took advantage of the HOD RBC model system. This system was elegantly created to leverage the HEL and OVA model antigens by coupling them to the blood group Duffy antigen (HOD) (30, 62–65). While early studies primarily employed the HOD system to study RBC-induced alloimmunization, this model has also been useful tool in studying the consequences of incompatible RBC transfusion. Initial investigations examining HOD RBC incompatible transfusion biology employed an anti-Duffy antibody known as Mima 29 (M29). Early data demonstrated that M29 was capable of inducing antigen loss and HOD RBC clearance, both of which required Fcγ receptors (66). Additional studies have shown that M29 can trigger AMIS (47), though recent findings indicate that the concentration of HOD RBCs may determine whether AMIS occurs or if there’s an enhanced immune response to HOD RBCs (46). Unlike M29, antibodies against HEL can bind to HOD RBCs but don’t cause significant RBC clearance (45, 56, 57, 67, 68). Instead, anti-HEL antibodies, either individually or in combination, can lead to the removal of the HEL antigen independent of Fcγ receptors (67, 68). This outcome is similar to the effects of polyclonal anti-HEL interaction with HEL observed in other experimental models (56, 57, 67). Additional investigations using different anti-Duffy antibodies have revealed that, like M29, these antibodies can also induce either AMIS or an amplified immune response (47, 48, 50). Distinct combinations of antibodies can enhance the likelihood of AMIS in general, including reversal of antibody-induced augmentation of RBC alloimmunization (45, 46, 69).

To investigate the temporal dynamics of antibody binding on the RBC surface, we sought to identify an antibody-antigen pair that could be studied without the confounding impact of antigen modulation or RBC clearance that would independently affect measurements of antibody binding. We selected the well-characterized M29 antibody in conjunction with the HOD model system, as M29 depends on Fcγ receptors for HOD RBC antigen modulation and clearance, and is available at concentrations suitable for direct labeling (66). This choice allowed us to directly examine antibody engagement dynamics and compare *in vivo* and *in vitro* binding patterns. Our data demonstrate that antibody binding to the HOD RBC surface is very dynamic and that while pre-incubating RBCs with antibody can reduce recognition, antibody pre-bound to HOD RBCs can rapidly dissociate in the presence of pre-administered antibody. These findings have implications for the mechanisms of AMIS, potential strategies to prevent hemolytic transfusion reactions, and dynamics of antibody binding *in vivo* relevant to antibody-based therapeutics.

## Materials and methods

### Mice

HOD RBC donor mice were generated and maintained as outlined previously (63). Fcer1g KO (FcγR KO) mice, 8-12 weeks old, were obtained from Taconic. Mice were housed at the Brigham and Women’s Hospital (BWH) Center for Comparative Medicine (CCM) as outlined previously (70). All studies were approved by the Mass General Brigham (MGB) Institutional Animal Care and Use Committee (IACUC).

### Antibodies and antibody labeling

MIMA-29 (M29), a mouse monoclonal anti-Duffy antibody and 2F4 and 4B7 mouse monoclonal anti-hen egg lysozyme (HEL) IgG antibodies, were generated as outlined previously (71, 72). Rabbit anti-ovalbumin (OVA) IgG polyclonal antibodies were obtained from Thermo Fisher Scientific. M29 was labeled using Alexa Fluor 647 (AF647) NHS ester or AF660 NHS ester (Thermo Fisher Scientific), using a similar protein labeling protocol as outlined previously (73–77). The labeled antibodies were purified from free dye using a PD-10 desalting column with Sephadex G-25 resin (Cytiva) (74). 2F4, 4B7, and anti-ovalbumin antibodies were biotinylated using sulfo-NHS-LC-biotin (Thermo Fisher Scientific). Free biotin was removed using a biotin removal column (Thermo Fisher Scientific).

### Examination of M29 antibody binding to HOD RBCs *in vitro* and *in vivo*

B6 RBCs and HOD RBCs were fluorescently labeled with lipophilic dye DiO and DiI, respectively, as described previously (56, 57, 60, 66, 78, 79). Using this approach, the survival of HOD RBCs labeled with DiI can be directly compared to the survival of DiO labeled B6 RBCs in the exact same recipient. HOD RBCs were coated with AF660-M29 by staining with a saturating solution for 30 minutes at 37°C. Then, unbound antibody was removed by washing with PBS. For *in vitro* experiments, HOD RBCs were incubated in the presence or absence of distinct antibody combinations as indicated in a polystyrene 96-well U-bottom plate at 37°C on a shaker. For *in vivo* experiments, FcγR KO mice served as recipients due to their inability to remove antibody-coated RBCs. Mice were passively administered 200 μg of AF647-M29 via the lateral tail vein, approximately 1 hour prior to transfusion. Mice were transfused via the lateral tail vein with a combination of 50uL of packed DiO B6 RBCs and 50uL of packed DiI HOD RBCs. At multiple timepoints, a blood sample was collected from the 96-well plate or the mouse tail vein.

### Flow cytometry

Following the acquisition of blood samples at the timepoints indicated, each sample was directly analyzed or washed and stained with the following: BV421 TER-119 (1:20), BV421 anti-mouse IgG (1:100), biotinylated anti-mouse C3 or C3b (1:100) followed by BV421 streptavidin (1:200), or biotinylated anti-HEL antibodies (1:100) followed by BV421 streptavidin (1:200), or biotinylated anti-OVA antibodies (1:100) followed by BV421 streptavidin (1:200) (46). Samples were analyzed using a 3 laser Cytek Northern Lights flow cytometer (22, 46). To compare binding, MFI values for M29 were normalized to maximal mean fluorescence intensity observed for each experiment.

### Data and statistical analysis

Flow cytometry data were analyzed using FlowJo, while statistics were performed using GraphPad Prism. Three or more groups were compared by one-way ANOVA with multiple comparisons by Tukey’s test, unless otherwise noted. p<.05 was the cut-off statistical for significance.

## Results

### Labeling and analysis of anti-Duffy antibody for equilibrium studies

Previous studies have demonstrated the ability of the anti-Duffy antibody, MIMA-29 (M29), to engage HOD RBCs and induce rapid clearance through a Fcγ receptor-dependent process (46, 66). However, in the absence of Fcγ receptors, no detectable RBC removal or changes in the RBC HOD antigen are observed, providing a possible approach to study the dynamics of antibody engagement following RBC exposure *in vivo* in the absence of RBC clearance or antigen modulation (66). Traditional antibody detection methods require removal of free antibody followed by detection of antibody engagement using a secondary anti-IgG antibody binding reagent, which could in theory create an additional variable of antibody detection efficiency using this approach. Furthermore, as secondary reagents can’t distinguish between distinct populations of the same antibody that are initially bound or free, examining dynamics between initially bound and free antibodies requires direct differential *a priori* labeling of each antibody population. As a result, we sought to develop a strategy capable of directly detecting antibody bound to RBCs without the need of secondary detection reagents. To this end, M29 was labeled with NHS esters of AF660 or AF647, allowing each antibody to be uniquely detected using spectral flow cytometry.

**Figure 1:**
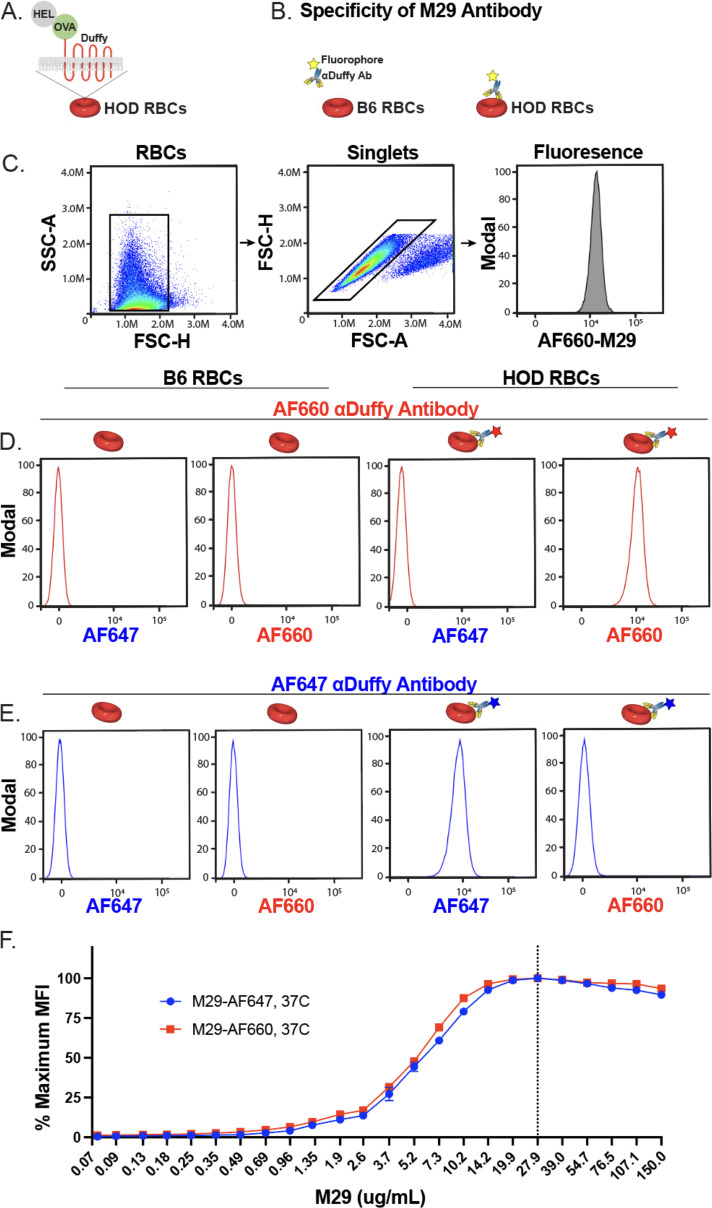
Alexa Fluor 660 or Alexa Fluor 647 labeled anti-Duffy antibodies exhibit distinct fluorescent patterns and similar binding characteristics toward HOD RBCs. **(A)** Schematic of HOD RBCs. **(B)** Schematic of binding evaluations of Alexa Fluor 660 (AF660-M29) or Alexa Fluor 647 (AF647-M29) anti-Duffy antibodies toward B6 or HOD RBCs. **(C)** Gating strategy used to identify RBC populations and binding profile following incubation with AF660-M29 or AF647-M29. **(D)** Binding profile of AF660-M29 shown as histograms toward B6 RBCs or HOD RBCs when using distinct spectral outputs to measure the relative fluorescence as indicated. **(E)** Binding profile of AF647-M29 shown as histograms toward B6 RBCs or HOD RBCs when using distinct spectral outputs to measure the relative fluorescence as indicated. **(F)** Examination of AF660-M29 or AF647-M29 binding to HOD RBCs over a range of concentrations as indicated plotted as maximal mean fluorescence intensity (MFI). Data are representative of 3 independent experiments.

To evaluate interactions of AF660-M29 with HOD RBCs, this antibody was incubated with HOD RBCs followed by assessment by flow cytometry (**Figures 1A–C**). As a control, B6 RBCs, which do not express the HOD antigen, were likewise incubated with AF660-M29 for comparison. AF660-M29 engagement of HOD RBCs was readily detected, while no binding was observed toward B6 RBCs (**Figure 1D**). To determine whether similar engagement could be appreciated following incubation with the alternatively labeled, AF647-M29, we similarly incubated either HOD RBCs or B6 RBCs with AF647-M29. Similar to AF660-M29, AF647-M29 bound to HOD RBCs, while appreciable binding to B6 RBCs did not occur (**Figure 1E**). Importantly, no fluorescent signal was detected for AF647 on AF660-M29 bound HOD RBCs or for AF660 on AF647 bound HOD RBCs, demonstrating that these fluorochromes can be adequately separated using this flow cytometric approach.

### Assessing the dynamics of M29 engagement of HOD RBCs *in vitro*

To assess whether either M29 label differentially influenced antibody engagement with HOD RBCs, we evaluated the binding of HOD RBCs by AF660-M29 or AF647-M29 across a broad range of concentrations. The analysis of each antibody across different concentrations revealed comparable binding profiles, indicating that both antibodies exhibited similar binding characteristics over the tested concentration range (**Figure 1F**). These findings suggest that while labeling of M29 allows direct detection of HOD RBCs, the labeling process does not significantly alter the overall binding profile of M29 to the HOD RBC target.

**Figure 2:**
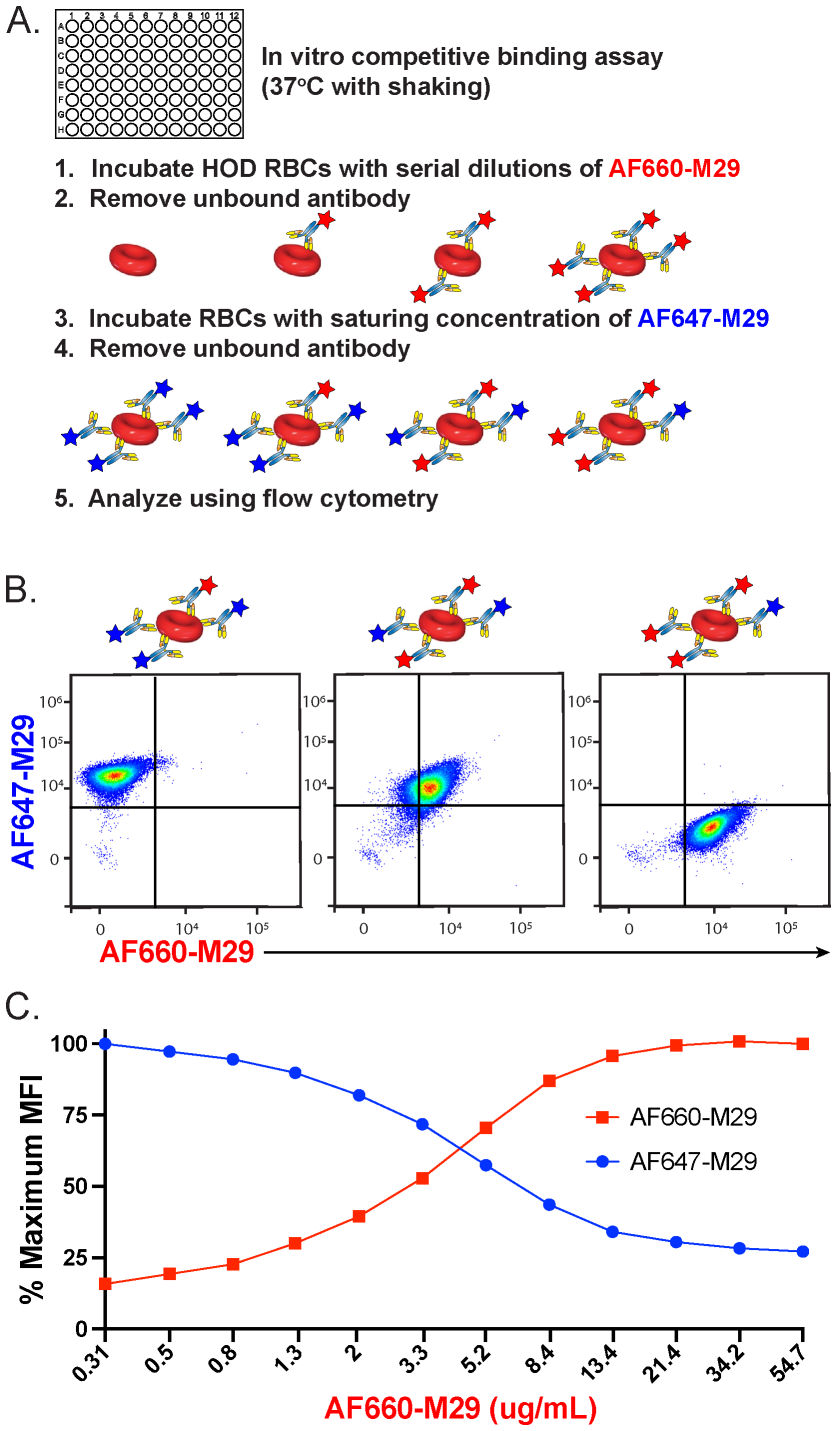
Preincubation of HOD RBCs with anti-Duffy antibody reduces additional binding by free antibody *in vitro*. **(A)** Schematic of the experimental approach used to examine the impact of prebound Alexa Fluor 660 (AF660)-M29 antibody on free Alexa Fluor 647 (AF647-M29) anti-Duffy antibody association to HOD RBCs over the course of 30 minutes. **(B)** Flow cytometric analysis of AF660-M29 or AF647-M29 toward HOD RBCs at distinct ratios of AF647-M29 to AF660-M29. **(C)** Quantification of binding by each antibody as a function of maximal mean fluorescence intensity (MFI) over a range of AF660-M29 concentrations as indicated followed by incubating with a saturating concentration of AF647-M29. Data are representative of 3 independent experiments.

To determine if preincubation with M29 can impact further engagement by M29 antibodies, HOD RBCs were incubated with a serial dilution of AF660-M29, followed by removal of unbound antibodies (**Figure 2A**). The AF660-M29 incubated RBCs were then exposed to a saturating concentration of AF647-M29 and analyzed using flow cytometry. Using this approach, as the concentration of AF660-M29 increased, its binding toward HOD RBCs likewise increased, while the levels of detectable AF647-M29 correspondingly decreased (**Figures 2B, C**). These findings suggest that different M29 fluorescent conjugates can be studied after engagement *in vitro*, that the distinctly labeled antibodies can be readily detected by flow cytometry, and that binding by one antibody can inhibit the binding of the other differentially labeled M29 antibody.

### Comparing the dynamics of M29 HOD RBC interactions *in vitro* and *in vivo*

**Figure 3:**
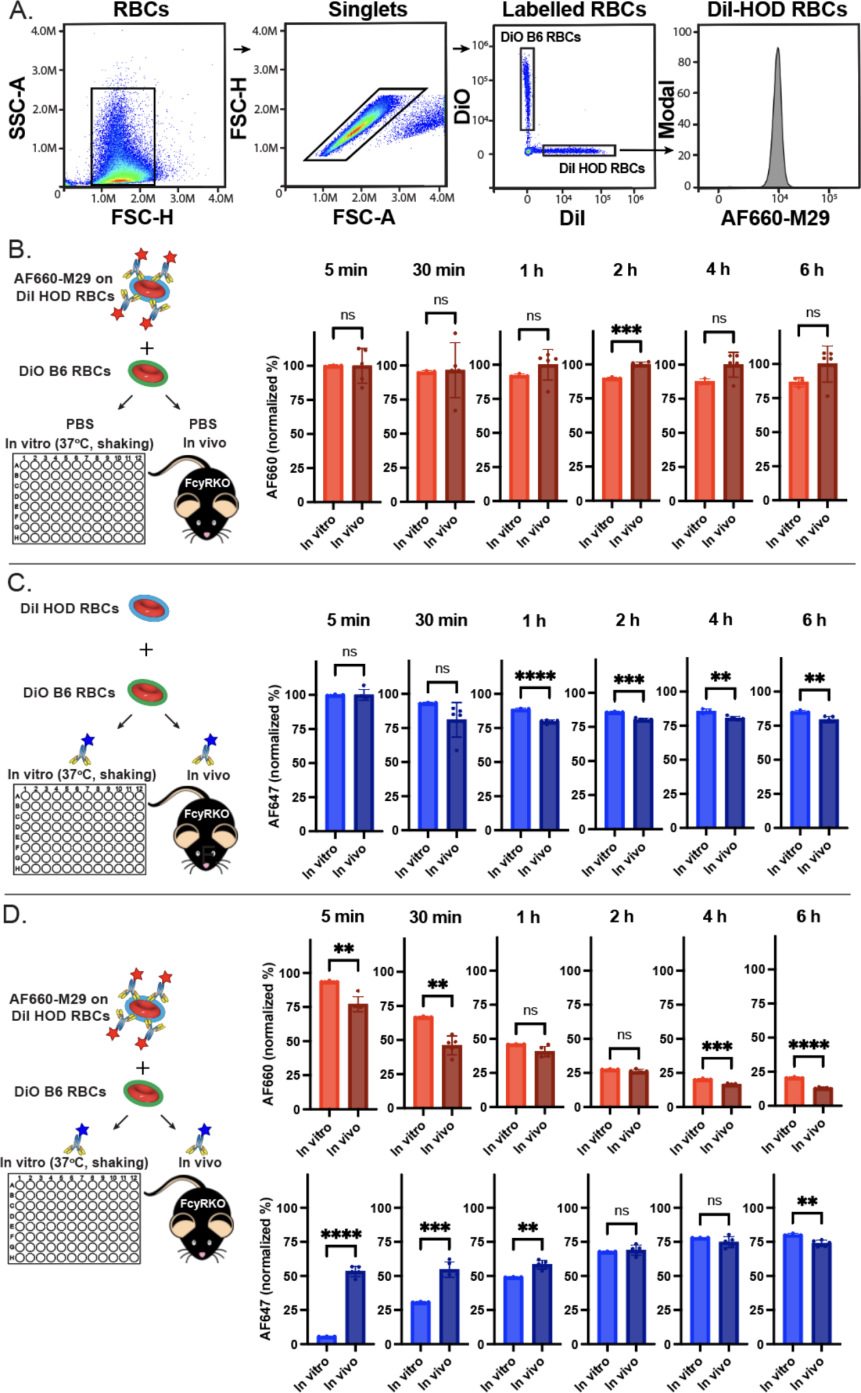
Free anti-Duffy antibodies rapidly associate with HOD RBCs even in the presence of prebound antibodies. **(A)** Flow cytometric gating strategy of differentially labeled HOD or B6 RBCs after *in vivo* or *in vitro* incubation. **(B)**. Examination of precoated Alexa Fluor 660 (AF660-M29) binding on HOD RBCs over the time points indicated *in vitro* and *in vivo*. **(C)** Examination of Alexa Fluor 647 anti-Duffy antibody (AF647-M29) following exposure of HOD RBCs to free anti-Duffy antibody over the time points indicated *in vitro* and *in vivo*. **(D)** Examination of AF660-M29 or AF647-M29 anti-Duffy antibodies binding to HOD RBCs following exposure of HOD RBCs precoated with AF660-M29 and with free AF647-M29 over the time periods indicated *in vitro* or *in vivo*. Data are representative of 3 independent experiments *P < .05; **P < .01; ***P < .001; ****P < .0001; ns, not significant.

To examine the dynamics of antibody engagement, we next defined the kinetics of antibody dissociation. This was achieved by preincubating HOD RBCs with saturating levels of AF660-M29 and subsequently assessing antibody binding over time following incubation *in vitro* or following transfusion of HOD RBCs into FcγR KO recipients *in vivo*. Using this approach, minimal change in AF660-M29 binding was observed within the first 30 minutes of analysis (**Figures 3A, B**). However, within 1 hour, a trend towards reduced levels of detectable antibody was observed *in vitro* compared to *in vivo*, with this difference reaching significance by 2 hours. This trend persisted beyond 2 hours, with antibody levels beginning to dissociate from HOD RBCs in some *in vivo* recipients, resulting in a relatively dispersed pattern of antibody binding. While this pattern was not significantly different from the levels of antibody engagement on HOD RBCs observed *in vitro*, the trend toward reduced levels of bound antibody was apparent on HOD RBCs *in vitro* when compared to *in vivo*. These findings indicate that M29 has a relatively slow dissociation rate, and that the rate of dissociation may be somewhat faster *in vitro* than *in vivo*.

We next sought to determine the on-rate of M29 toward HOD RBCs *in vitro* and *in vivo*. To accomplish this, HOD RBCs were introduced to saturating levels of AF647-M29, followed by analysis of antibody engagement (**Figure 3C**). Within 5 minutes of exposure to AF647-M29, the levels of AF647-M29 *in vitro* and *in vivo* achieved the same saturating levels observed following titration of M29 over a range of concentrations. However, within 30 minutes, these levels began to decline, with the levels observed on HOD RBCS obtained from mice *in vivo* trending toward an accelerated rate of antibody loss when compared to the same HOD RBCs *in vitro*. This same pattern continued, with the levels of M29 detected on the surface of HOD RBCs in both settings continuing to decline through 1 hour, whereupon these differences appeared to stabilize with the levels observed *in vivo* persisting at a slightly lower level than observed *in vitro* thereafter.

To assess the potential impact of prior antibody engagement on the on-rate of free M29 associated with HOD RBCS, we next preincubated HOD RBCs with AF660-M29, followed by evaluation of AF660-M29 and AF647-M29 binding once exposed to free AF647-M29 *in vitro* and *in vivo* (**Figure 3D**). Exposure of HOD RBCs previously coated with AF660-M29 to AF647-M29 resulted in a gradual increase in AF647-M29 over time, with a low level of binding being detected at 5 minutes following incubation *in vitro* that increased to stable levels around 4 hours post exposure. In contrast, introduction of AF660-M29 precoated HOD RBCs to free AF647-M29 *in vivo* resulted in a rapid change in the dynamics of engagement of HOD RBCs. In contrast to the findings observed *in vitro*, significant binding by AF647-M29 could be detected as early as 5 minutes following exposure *in vivo*. However, following this initial wave of AF647-M29 binding, the rate of additional antibody binding engagement decreased, with levels of AF647-M29 binding increasing over time at a slower rate. In settings where increased levels of AF647-M29 was observed, a corresponding decrease in AF660-M29 binding was found (**Figure 3D**), suggesting displacement of bound AF660-M29 with AF647-M29 over time. Although AF647-M29 engagement was slower *in vitro*, it achieved comparable levels of antibody binding to those observed *in vivo* within 2 hours. Despite minor differences in binding, the antibody levels remained relatively consistent over the remainder of the analysis.

To address the possibility that observed differences in association and dissociation rates between *in vitro* and *in vivo* conditions might be attributed to the specific fluorochromes used in the antibody combinations, we next conducted the same experiments with reversed fluorochrome pairings. We repeated the same experimental protocol, this time preincubating HOD RBCs with AF647-M29 and examining binding by AF660-M29 initially in solution. The results showed comparable trends in dissociation rates for both *in vitro* and *in vivo* conditions, with similar association and equilibration patterns being observed when exposing HOD RBCs to AF660-M29 in both situations (**Supplementary Figure 1**). While the rate of prebound AF647-M29 dissociation *in vitro* appeared to be slightly increased when HOD RBCs were incubated in the absence of any other antibody (**Supplementary Figure 1A**), the binding of AF660-M29 *in vivo* following initial engagement appeared to retain at a slightly higher level than observed previously (**Supplementary Figure 1B**). Most notably, the on-rate of free AF660-M29 binding to AF647-M29 precoated HOD RBCs occurred at a similar rate as observed when examining AF660-M29 precoated cells exposed to free AF647-M29. Likewise, the off-rate of AF647-M29 in the presence of free AF660-M29 exhibited a similar pattern to that observed when HOD RBCs precoated with AF660-M29 were exposed to free AF647-M29 (**Supplementary Figure 1**). These findings suggest that while subtle differences occurred, the observed differences in antibody binding kinetics between *in vitro* and *in vivo* settings were not likely significantly influenced by the choice of fluorochromes used in the antibody combinations.

### Alterations in antibody engagement do not reflect RBC clearance or antigen modulation

**Figure 4:**
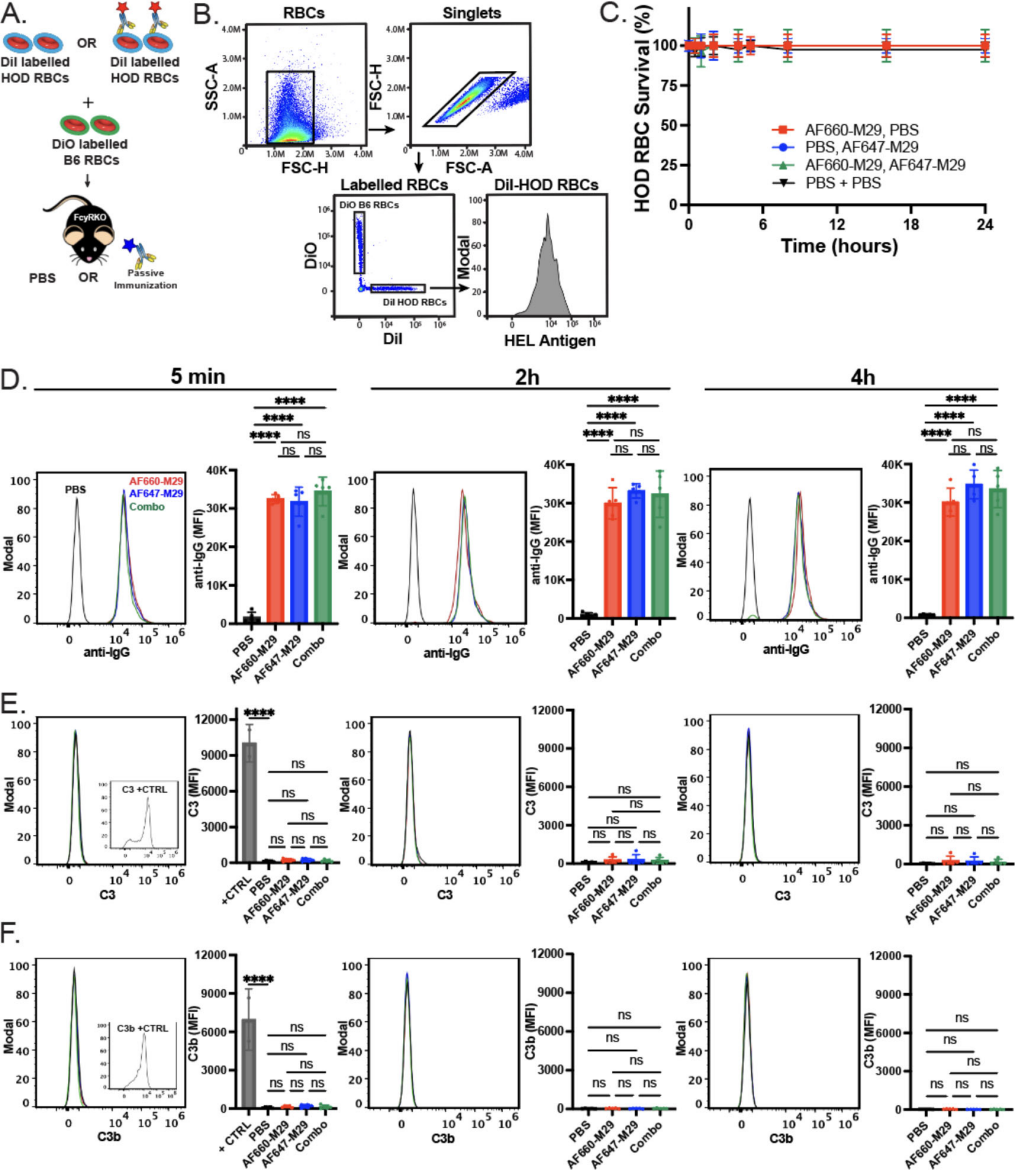
Anti-Duffy antibody engagement fails to induce detectable changes in HOD RBC survival or complement deposition on the HOD RBC surface. **(A)** Schematic of the experimental approach. **(B)** Flow cytometric gating strategy of HOD RBCs following transfusion of differentially labeled HOD or B6 RBCs for analysis of HOD RBC survival, total antibody binding, complement deposition or detection of antigen levels. **(C)** Survival of HOD RBCs in the presence of Alexa Fluor 660 anti-Duffy antibodies (AF660-M29), Alexa Fluor 647 anti-Duffy antibodies (AF647-M29), both or neither (PBS) as indicated. **(D)** Examination of bound antibody levels as histograms and mean fluorescent intensity (MFI) values at the time points indicated post-transfusion in the presence of AF660-M29, AF647-M29, both or neither as indicated. **(E)** Examination of C3 levels as histograms and MFI values at the time points indicated post-transfusion in the presence of AF660-M29, AF647-M29, both or neither as indicated. **(F)** Examination of C3b levels as histograms and MFI values at the time points indicated post-transfusion in the presence of AF660-M29, AF647-M29, both or neither as indicated. Data are representative of 3 independent experiments. ****P < .0001; ns, not significant.

The primary goal of using FcγR KO recipients was to eliminate the potential confounding effect of antibody-mediated clearance on the analysis of overall antibody engagement with HOD RBCs *in vivo*. However, as the current assessment employed fluorescently labeled M29, we next sought to assess whether antibody engagement impacted HOD RBC survival when exposed to AF660-M29, AF647-M29, or both. To better identify the possible removal of HOD RBCs in each setting, two distinct labeling methods were employed. HOD RBCs were marked with DiI, while the HOD-negative B6 RBCs were labeled with a fluorescently distinct dye, DiO. This dual-labeling approach provided an internal antigen negative control, allowing for a direct comparison of RBC survival rates within each experimental condition. Despite the clear presence of antibody on the cell surface in each scenario, no difference in HOD RBC survival was observed, consistent with previous findings (**Figures 4A–D**). These data suggest that exposure of HOD RBCs to labeled M29 did not significantly affect HOD RBC survival and that differences in antibody engagement observed over time therefore did not reflect detectable antibody-induced alterations in RBC survival.

The inability of M29 to impact HOD RBC survival is consistent with the notion that M29-mediated clearance of HOD RBCs occurs through an Fcγ receptor-dependent process. However, as IgG antibodies can fix complement and complement could possibly mask or otherwise impact antibody engagement of the HOD antigen *in vivo* irrespective of its involvement in RBC clearance, which in turn could influence the dynamics of M29 binding, we examined complement component 3 (C3) levels on the HOD RBC surface in the presence or absence of combinations of differentially labeled M29. No significant difference in C3 or C3b levels could be detected on HOD RBCs in the absence of M29 or following exposure to AF660-M29, AF647-M29 or both, strongly suggesting that C3 deposition on the RBC surface did not impact the levels of M29 engagement observed in each setting *in vivo* (**Figures 4E, F**).

**Figure 5:**
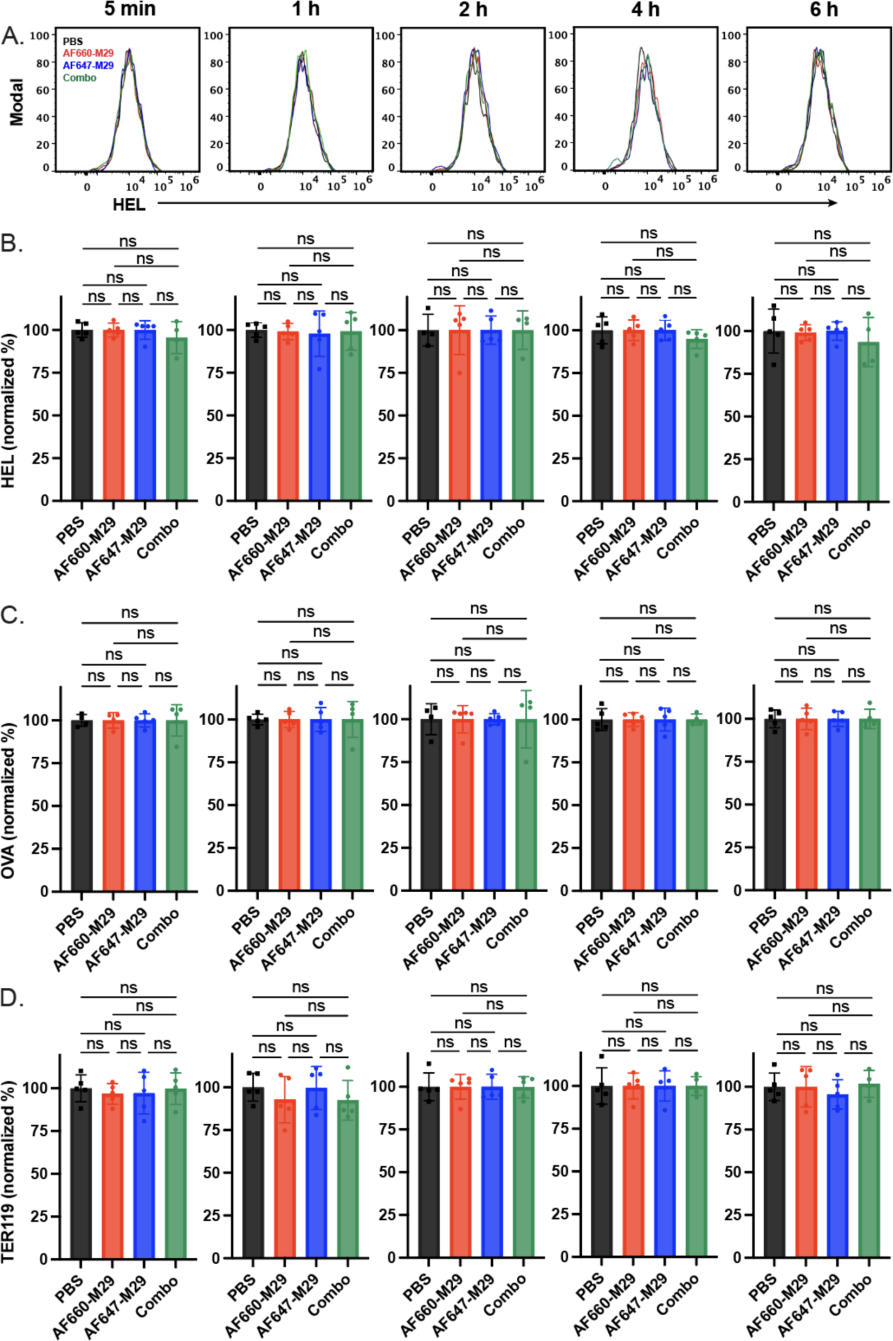
Anti-Duffy antibody engagement fails to induce detectable changes in the levels of HEL, OVA, and Ter119. **(A)** Histograms of HEL antigen levels as measured by flow cytometry on HOD RBCs in the presence of Alexa Fluor 660 anti-Duffy antibodies (AF660-M29), Alexa Fluor 647 anti-Duffy antibodies (AF647-M29), both or neither (PBS) as indicated. **(B)** Quantification of HEL antigen levels as measured by flow cytometry on HOD RBCs in the presence of AF660-M29, AF647-M29, both or neither as indicated. **(C)** Quantification of OVA antigen levels as measured by flow cytometry on HOD RBCs in the presence of AF660-M29, AF647-M29, both or neither as indicated. **(D)** Quantification of Ter119 staining as measured by flow cytometry on HOD RBCs in the presence of AF660-M29, AF647-M29, both or neither as indicated. Data are representative of 3 independent experiments. ns, not significant.

Prior studies not only demonstrated that M29 can induce HOD RBC clearance, but also antigen modulation, wherein a reduction in the HOD antigen can be detected on the surface of HOD RBCs following antibody engagement. As the levels of antigen would be predicted to impact the amount of detectable antibody on the cell surface, we next examined whether exposure of HOD RBCs to AF660-M29, AF647-M29, or both in any way impacted the levels of HOD antigen, by examining HEL or OVA portions of the HOD surface antigen. Using this approach, no difference in HEL or OVA was detected when comparing HOD RBCs transfused in the absence of M29 to HOD RBCs exposed to AF660-M29, AF647-M29, or both (**Figures 5A–C**). Similarly, no difference in the RBC surface marker Ter119 was likewise detected in the presence or absence of M29, strongly suggesting that differences in M29 engagement over time did not reflect detectable differences in the levels of the HOD target antigen on the RBC surface (**Figure 5D**).

## Discussion

RBCs serve as an attractive vehicle to study the dynamics of antibody-antigen interactions on the surface of a cell *in vitro* and *in vivo*. In contrast to white blood cells, mature RBCs do not have a nucleus, endoplasmic reticulum, Golgi apparatus, or endolysomal machinery and, therefore, do not change antigen expression in a similar manner to what may occur on nearly every other cell (80). The relatively static residence of proteins on the surface of an RBC can prevent cell-intrinsic changes in surface levels of expression from significantly influencing measurements of antibody engagement over time. Furthermore, RBCs do not possess phagocytotic activity and, therefore, are unlikely to possess the ability to actively remove antibody bound to their surface once engaged (81). As a result, RBCs can serve as a useful chassis when seeking to evaluate antibody-antigen interactions at the cell surface in the absence of other variables that would normally confound the interpretation of antibody engagement on the cell surface over time. As such, RBCs provide an attractive vehicle to study the kinetics of antibody interactions with cell surface antigens. Using RBCs, IgG antibodies were found to rapidly equilibrate on the RBC surface *in vitro* and *in vivo*. However, the ability of free antibodies to equilibrate with antibody bound HOD RBCs *in vivo* was more pronounced and an unexpected finding in this study. These results suggest that displacement of bound antibody, even if dissociation rates are thought to relatively slow, can be rapid, with implications in a wide variety of settings in which antibodies are used to block epitope recognition.

The dynamics of antibody-antigen interactions play a crucial role in various immunological and therapeutic contexts. Efficient binding of antibodies to pathogens is essential for facilitating microbial clearance through a range of immune mechanisms that leverage antibody effector functions, including complement activation and Fc receptor-mediated processes (82–84). Antibody-mediated neutralization of toxins or viral attachment proteins forms the basis of many vaccine strategies, requiring engaged antibodies to remain bound with sufficient affinity to prevent effective engagement of host receptors (85–88). In the realm of antibody-based therapeutics, effective target binding serves as a mechanism to neutralize the bioactivity of soluble targets or remove bound cells, depending on the specificity of the therapeutic antibody (89). While monoclonal antibody-based therapies have significantly expanded the use of biologics for therapeutic purposes, the use of anti-RhD antibodies to prevent alloimmunization against RhD represents one of the earliest applications of antibodies with defined specificity for prophylactic clinical outcomes (90, 91). A compelling hypothesis suggests that passively administered polyclonal anti-RhD antibodies may prevent the initiation of alloimmunization by masking RhD epitopes on the cell surface, thereby hindering effective immune recognition and response (92, 93). While this mechanism may be relevant in antibody-mediated immunosuppression, the dynamics of antibody interactions observed on the cell surface suggest that the consequences of antibody engagement may be more complex than previously appreciated. The importance of antibody-antigen engagement extends beyond pathogen neutralization and therapeutic applications. The use of RBCs pre-coated with antibodies lacking effector functions has been proposed as a potential strategy to prevent hemolytic transfusion reactions (94–96). These strategies highlight the versatility of antibody-antigen interactions in achieving desired clinical outcomes.

Despite the extensive use of antibodies in therapeutics and their critical role in immune responses, antibody-antigen interaction dynamics in living systems remain incompletely understood. This stems from challenges in studying these interactions *in vivo* without confounding factors and the varied distribution of antigen targets across body compartments (97). While RBC alloantigens likely evolved under diverse selective pressures, including infectious diseases (73–77), and can complicate transfusion management and pregnancy, allogeneic RBCs offer a unique model for investigating antibody-antigen interactions *in vivo*. Furthermore, although alloimmunization is not exclusive to RBCs and can affect platelet transfusions and recombinant protein therapies (98–102), RBC alloantigens present distinct advantages for studying antibody-antigen kinetics in living organisms, where the unique properties of RBCs provide an opportunity to examine these interactions in a well-defined system.

The HOD model system can potentially shed light on broader principles of antibody-antigen engagement applicable to other therapeutic and immunological scenarios. In addition to not dividing or synthesizing new antigens, RBCs primarily circulate within the vascular system, which allows for easy and repeated sampling over time. While a variety of factors influence antibody effector function (84), these collective properties make RBCs an ideal platform for assessing the dynamics of antibody-antigen interactions in a living system. Equally important, certain antibodies, such as M29, do not induce RBC clearance or antigen modulation in the absence of Fcγ receptors (66). This characteristic enables studies to isolate changes in antibody detection specifically to alterations in the kinetics of antibody-antigen interactions, rather than confounding factors such as RBC removal or antigen modulation (45, 46, 48–58). By leveraging these unique properties of RBCs and non-clearing antibodies, the temporal dynamics of antibody-antigen interactions *in vivo* can be examined.

The results of the present study suggest that antibody-antigen interactions, at least on the RBC surface, may be more dynamic that previously appreciated. Hypotheses that have evoked steric hindrance as a mechanism whereby passively administered antibodies can sterically hinder recognition of a target antigen would be predicted to rely on relatively stable antibody engagement following antigen recognition on the cell surface (92, 93). Similarly, the concept of pre-coating RBCs with afunctional antibodies that possess the ability to mask antigens (94–96), thereby preventing recipient antibody engagement of the target, would likewise be predicted to require relatively stable antibody interactions the face of additional antibodies if RBCs are to be protected following an incompatible transfusion. However, the dynamic nature of these interactions might involve rapid association and dissociation events, allowing for a more complex interplay between different antibodies and antigens on the cell surface. Indeed, the ability of free antibody to engage antigen, even in the presence of precoated antibody, suggests that the dynamics of antibody-antigen interactions may be quite fluid, raising the possibility that other mechanisms and overall strategies, beyond steric hindrance, may be considered when seeking to understand the concepts like AMIS or design approaches to prevent hemolytic transfusion reactions following an incompatible RBC transfusion.

The observed rate of free antibody binding to pre-coated HOD RBCs surpassed the rate of dissociation of pre-bound antibodies in the absence of free antibodies and suggests that dissociation rates may not always predict the ability of bound antibody to prevent epitope recognition. This phenomenon may be attributed to several factors. One plausible explanation is that when an antibody is bound through both Fabs, after partial dissociation of one bound Fab arm, the engagement of free antibodies prevents the re-association of the previously bound Fab, resulting in only one Fab remaining bound (103, 104). In this scenario, as free antibodies initially bind, the pre-bound and associating antibodies may reach a transition state where only one Fab is bound at a time, preventing the unbound Fab arm from quickly reassociating. Consequently, the likelihood of the other Fab binding to adjacent epitopes is reduced due to possible occupancy of adjacent epitopes by newly exposed antibodies in solution, reducing the overall strength of the antibody bound to the cell surface. Conversely, in the absence of competing free antibodies, the dissociated Fab domains of the pre-bound antibody are more likely to re-associate with the same antigen from which they just uncoupled (103). Since it is challenging to distinguish between antibody engagement through both Fab arms or one Fab using flow cytometry, and these associations and dissociations are likely in a constant state of equilibrium, the static read-out observed using flow cytometry merely indicates that an antibody is bound. However, when an antibody labeled with a distinct fluorochrome is present, the effects of these competing interactions can be observed.

The intricate interplay of antibody-antigen interactions and the potential influence of competitive binding are consistent with established biochemical principles governing antibody affinity and avidity (105). Unlike the classic positive cooperativity observed in systems such as hemoglobin-oxygen binding, where the engagement of one site directly alters the intrinsic affinity of other sites (106), antibody-antigen interactions typically do not exhibit this phenomenon. Instead, the microscopic equilibrium constants for each Fab arm are generally considered to remain stable irrespective of whether one Fab is engaged with a cognate antigen (103, 104). As a result, the apparent cooperativity in antibody binding likely reflects an associative mechanism in contrast to classic allostery. When one Fab engages its target, the effective concentration of the second arm increases dramatically in the vicinity of the cell surface where additional epitopes lie. This spatial proximity enhances the probability of a second binding event, contributing to the overall avidity of the interaction. Furthermore, bivalent binding provides a kinetic advantage; if one Fab arm temporarily dissociates, the continued attachment of the other arm significantly increases the likelihood of rapid reassociation (103, 104). However, this binding dynamic can be disrupted in the presence of competing antibodies. When one Fab arm disengages from its epitope, free competing antibodies may occupy the site, potentially preventing the rapid reassociation that would otherwise occur. This competitive binding scenario underscores the importance of considering both affinity and avidity in antibody-antigen interactions, particularly in therapeutic settings (103, 104). The interplay between these factors - bivalent binding, spatial proximity effects, and competitive interactions likely contributes to the complex and dynamic nature of antibody-antigen engagement.

As with any study, the present approach is not without limitations. As the goal of this study was to define the dynamics of antibody-antigen interactions, this study employed a model of HOD RBC transfusion in the presence or absence of differentially labeled pre-coated and free antibody to explore the kinetics of these associations. It is possible that the fluorochrome used for direct assessment of antibody binding may subtly influence antibody-antigen interactions and overall antibody behavior. To investigate this possibility, we conducted experiments with reversed antibody combinations, both free in solution and bound to HOD RBCs, to determine if the antibody label could affect antibody binding dynamics. Our results demonstrated that although minor alterations were detected, overall patterns remained consistent. Importantly, the distinct outcomes observed *in vivo* and *in vitro* when precoated HOD RBCs were exposed to unbound antibodies in solution, continued to be evident regardless of the specific antibody combinations used. These results underscore the importance of examining antibody dynamics *in vivo*, as they may differ from *in vitro* observations. Slight differences in the fluorochrome itself could result in subtle changes in antibody characteristics. Future studies may benefit from alternative conjugation methods to obtain more precise measurements of antibody-antigen dynamics.

Whether the dynamics observed here reflect similar changes in antibody binding that may occur following engagement by a B cell receptor (BCR) remain unknown. As no BCR transgenics currently exist for M29, this type of study is currently not feasible. While there are BCR transgenics for the HEL portion of HOD (107), prior studies demonstrated that anti-HEL antibodies induce removal of HEL in the absence of Fcγ receptors or complement (67), preventing the present strategy from being employed to examine the dynamics of antibody-antigen interactions using HEL as the target in the absence of antigen modulation. As a result, the use of anti-HEL antibodies for studying antibody-antigen dynamics on RBCs can pose challenges due to their ability to remove antigens in the absence of Fcγ receptors, making it difficult to distinguish between antibody dissociation and antigen removal. The M29 antibody in the HOD RBC model system offers a unique opportunity to study these dynamics without causing RBC removal or antigen modulation in Fcγ receptor KOs. While further research is needed to determine if these findings apply to other antibodies, such studies will require similar models where antibody engagement does not lead to RBC clearance or antigen modulation.

## Data Availability

The raw data supporting the conclusions of this article will be made available by the authors, without undue reservation.
