# Supplementary material for: Dynamics of antibody engagement of red blood cells in vivo and in vitro
Source: Front Immunol. 2024 Nov 28;15:1475470. doi: 10.3389/fimmu.2024.1475470 (PMC11634868; doi:10.3389/fimmu.2024.1475470)
Supplement: Supplementary file 1 [file DataSheet1.pdf]

Supplemental Figure 1

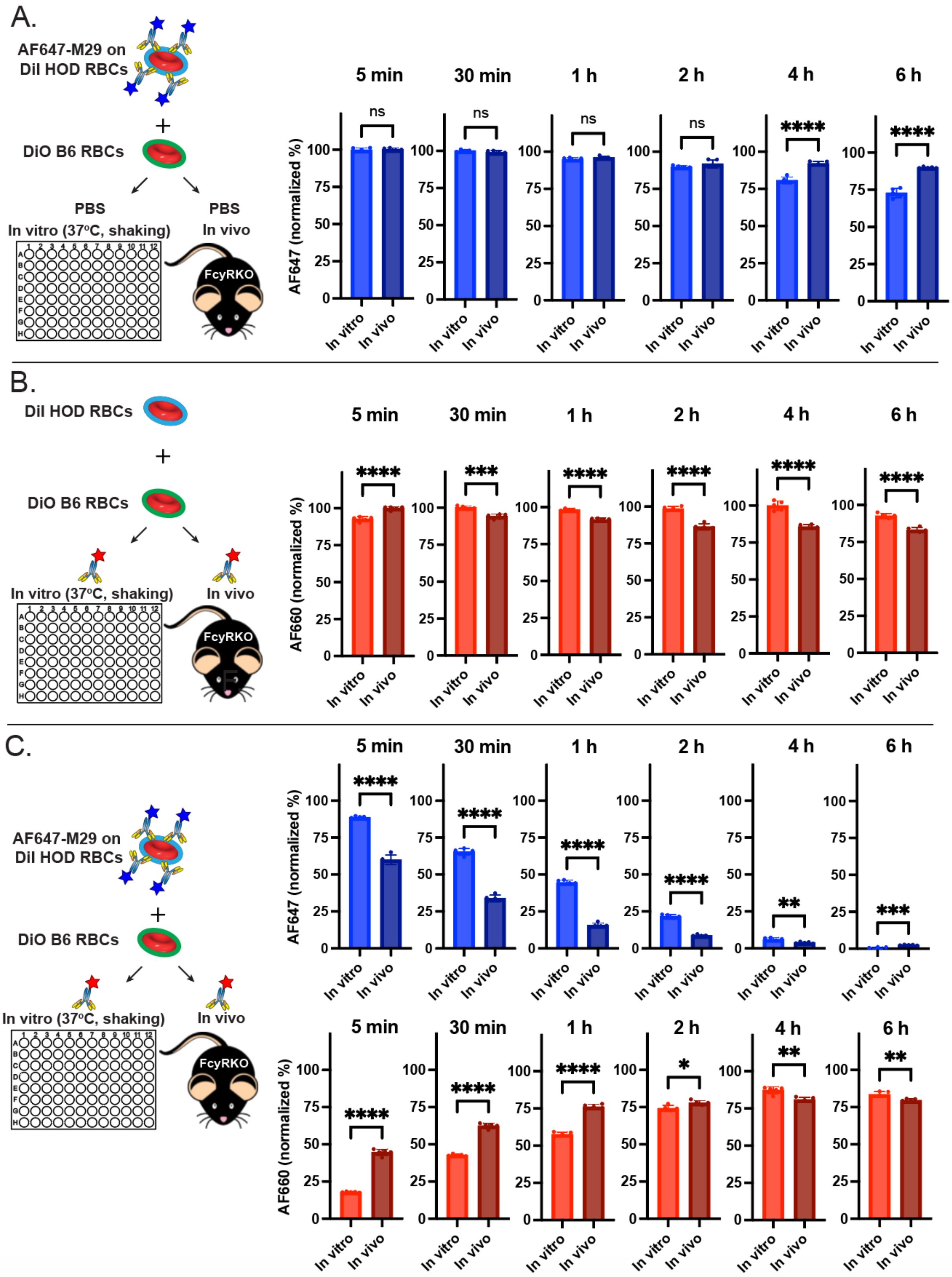

Supplemental Figure 1: Anti-Duffy antibody association and dissociation on HOD RBCs.

A). Examination of precoated HOD RBCs with Alexa Fluor 647 (AF647-M29) over the time points indicated *in vitro* and *in vivo*. B) Examination of Alexa Fluor 660 anti-Duffy antibody (AF660-M29) binding following exposure of HOD RBCs to free anti-Duffy antibody over the time points indicated *in vitro* and *in vivo*. C) Examination of AF647-M29 or AF660-M29 anti-Duffy antibodies binding to HOD RBCs following exposure of HOD RBCs precoated with AF647-M29 and with free AF660-M29 over the time periods indicated *in vitro* or *in vivo*. Data are representative of 2 independent experiments.
